# Construction of a Tumor Immune Microenvironment-Related Prognostic Model in BRAF-Mutated Papillary Thyroid Cancer

**DOI:** 10.3389/fendo.2022.895428

**Published:** 2022-06-08

**Authors:** Yuxiao Xia, Xue Jiang, Yuan Huang, Qian Liu, Yin Huang, Bo Zhang, Zhanjun Mei, Dongkun Xu, Yuhong Shi, Wenling Tu

**Affiliations:** ^1^ Department of Nuclear Medicine, The Second Affiliated Hospital of Chengdu Medical College, China National Nuclear Corporation 416 Hospital, Chengdu, China; ^2^ School of Bioscience and Technology, Chengdu Medical College, Chengdu, China

**Keywords:** BRAF, papillary thyroid cancer, prognostic model, nomogram, tumor-infiltrating immune cells

## Abstract

BRAF mutation is a representative oncogenic mutation, with a frequency of 60% in papillary thyroid carcinoma (PTC), but the reasons for the poor prognosis and more aggressive course of BRAF-mutated PTC are controversial. Tumor immune microenvironment (TIME) is an essential factor permitting the development and progression of malignancy, but whether TIME participates in the prognosis of BRAF-mutated PTC has not yet been reported. The primary goal of the present study was to provide a comprehensive TIME-related prognostic model to increase the predictive accuracy of progression-free survival (PFS) in patients with BRAF-mutated PTC. In this study, we analyzed the mRNA-seq data and corresponding clinical data of PTC patients obtained from the TCGA database. By calculating the TIME scores (immune score, stromal score and ESTIMATE score), the BRAF mutation group (n=237) was dichotomized into the high- and low-score groups. By functional analysis of differentially expressed genes (DEGs) in different high/low score groups, we identified 2 key TIME-related genes, *HTR3A* and *NIPAL4*, which affected PFS in BRAF-mutated PTC. A risk scoring system was developed by multivariate Cox analysis based on the abovementioned 2 TIME-related genes. Then, the BRAF-mutated cohort was divided into the high- and low-risk groups using the median risk score as a cutoff. A high risk score correlated positively with a higher *HTR3A/NIPAL4* expression level but negatively with PFS in BRAF-mutated PTC. Ultimately, a nomogram was constructed by combining risk score with clinical parameter (Tumor stage), and the areas under the ROC curve (AUCs) of the nomogram for predicting 1-, 3- and 5-year PFS were then calculated and found to be 0.694, 0.707 and 0.738, respectively, indicating the improved accuracy and clinical utility of the nomogram versus the risk score model in the BRAF-mutated PTC cohort. Moreover, we determined the associations between prognostic genes or risk score and immune cell infiltration by two-way ANOVA. In the high-risk score, high HTR3A expression, and high NIPAL4 expression groups, higher infiltration of immune cells was found. Collectively, these findings confirm that the nomogram is effective in predicting the outcome of BRAF-mutated PTC and will add a spatial dimension to the developing risk stratification system.

## Introduction

Malignant thyroid tumors are the most prevalent type of endocrine tumor (1). Papillary thyroid cancer (PTC) is the most frequent pathological type of thyroid malignancy, constituting approximately 80-85% of total reported cases. The prognosis of PTC is usually excellent, with a 5-year survival rate of >95% and a 10-year survival rate of >90% when treated with radioiodine ablation and/or revision surgery (2). However, 10-15% of patients with PTC usually present with aggressive tumor behavior and poor prognosis, with a stable recurrence rate of nearly 30% after 30 years of follow-up (3). In multiple studies, BRAF mutation is considered a risk factor associated with less favorable clinicopathological characteristics and a more aggressive course (4–7). BRAF mutation is a representative oncogenic mutation (8–10), found in 60% of PTC cases (11). The mechanism by which it affects the course of tumor aggressiveness is thought to be as follows: BRAF encodes B-RAF, a cytoplasmic serine/threonine kinase (STK) that is involved in constitutive activation of the RAF-MEK-ERK signaling cascade, thereby inhibiting apoptosis and promoting cell proliferation, which ultimately drives tumor growth (9). However, the possible relationship between BRAF mutation and aggressive behavior in PTC is controversial. The latest ATA recommendations do not routinely use BRAF status for initial risk stratification in PTC, suggesting that BRAF mutation is not yet a single, independent predictor. Therefore, we urgently need to develop a novel prognostic signature with the goal of improving efficiency in the management of PTC patients with BRAF mutation.

It is generally known that the tumor immune microenvironment (TIME) plays a key role in the disease course in cancer. The TIME is a complex ecosystem consisting of various kinds of immune cells (such as neutrophils, lymphocytes, mast cells and macrophages) and soluble mediators (chemokines, growth factors and cytokines). Its components interact with each other through autocrine and paracrine mechanisms as well as signaling pathway activation to inhibit immune surveillance and confer chemoresistance, thereby stimulating tumor invasion and metastasis (12–14). In addition to some clinical risk factors, such as the maximum diameter of the primary tumor, the number of tumors, thyroid capsule invasion status, and number of cervical lymph node metastases, one possible mechanism for the progression of PTC is the alteration of TIME status (15). Some studies have demonstrated that upregulation of TAMs, MCs, DCs, chemokines (such as *CXCL8*, *CXCL10*, *CXCL12*, *CXCL16* and *CXCL20*) and transcription factors (such as *NF-κB*, *HLF, HIF* and *Runx2*) in the TIME can promote the invasion of thyroid carcinoma cells (16–23). Recently, Rujia Qin et al. constructed an immune-related prognostic model containing 8 PTC progression-related genes (*ULBP2, S100A5, LTF, PLXNA4, FAM3B, GIPR, RORB* and *TGFBR*) (24), suggesting that TIME-related genes may affect the clinical outcome of PTC by remodeling the TIME. Although several studies have examined the association between TIME-related genes and PTC prognosis, few studies have focused on PTC with BRAF mutation.

Given that the burden of BRAF-mutated PTC is large and still rising, substantial efforts have been devoted to optimizing the prognostic prediction system and management strategies for this cancer (24–29). However, many risk classification systems, such as the American Thyroid Association (ATA) risk stratification and TNM staging, have failed to adequately predict the prognosis of individual patients (30). Therefore, exploration and screening of TIME-related prognostic models in BRAF-mutated PTC will likely be a future breakthrough.

**Figure 1:**
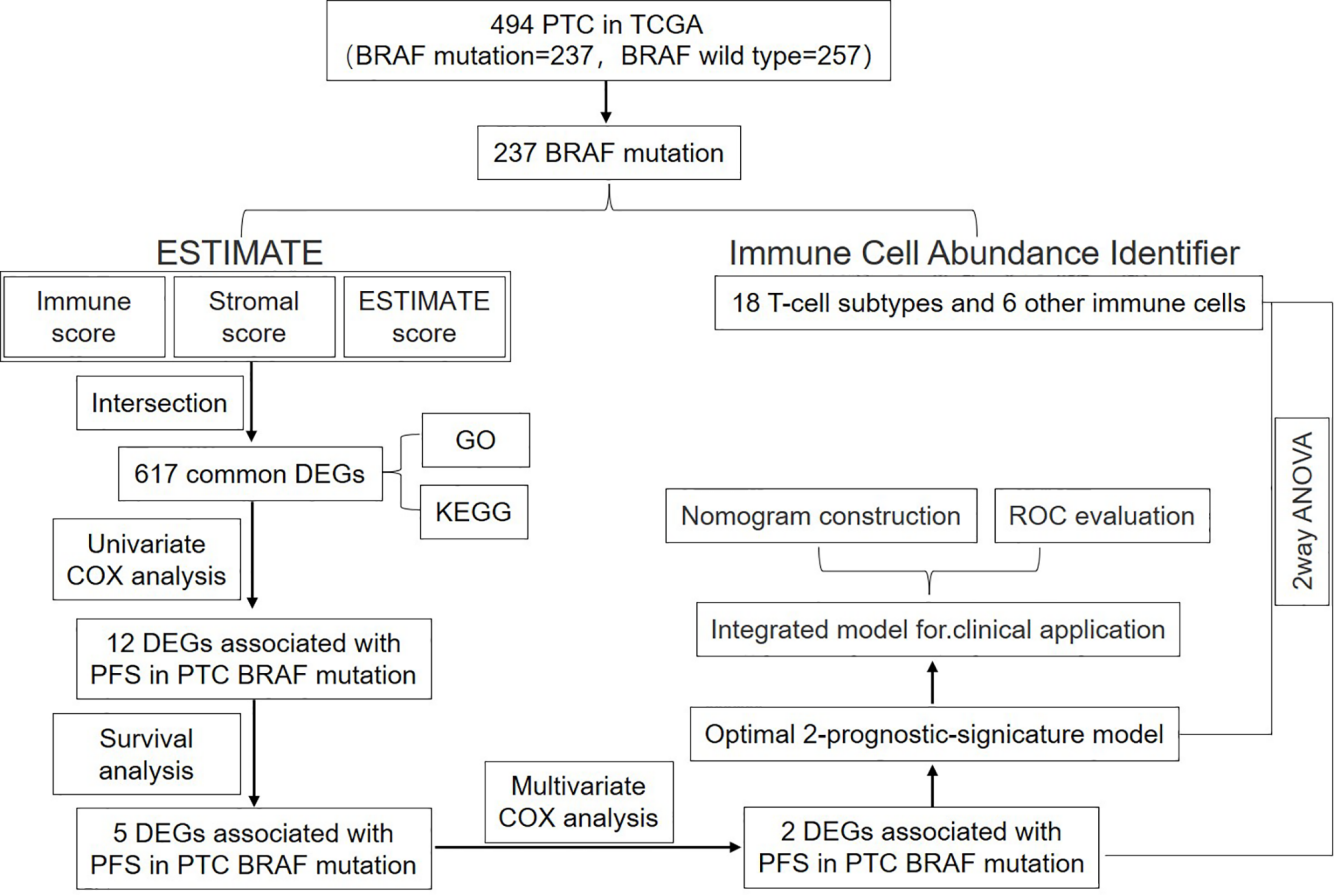
Analysis workflow of this study.

In this study, in order to verify the role of TIME-related genes in the prognosis of BRAF-mutated PTC and its relationship with TIME, mRNA-seq samples and corresponding clinical data from patients with BRAF-mutated PTC in the TCGA database were analyzed by bioinformatic methods. Based on functional analysis of DEGs with three TIME scores (immune score, stromal score and ESTIMATE score), the key TIME-related genes affecting the PFS of BRAF-mutated PTC were identified. The TIME-related genes with prognostic significance were integrated into a multivariate prognostic Cox model to calculate the risk score. By integrating various clinical parameters with the risk score, a nomogram was developed for PFS prediction in BRAF-mutated PTC. Ultimately, the reliability of the predictions was assessed by tROC curve analysis. In addition, we calculated the associations between prognostic genes or risk score and immune cell infiltration by two-way ANOVA to investigate the significance of tumor-infiltrating immune cells (TICs) in predicting prognosis. The workflow is shown in **Figure 1**.

## Materials and Methods

### Characteristics of PTC Datasets

The mRNA-seq data, somatic mutation data and clinical information of PTC patients (n=494) were downloaded from the TCGA Genomic Data Commons (GDC) (https://portal.gdc.cancer.gov/repository) portal. The clinical data included sex, age, radiotherapy history, metastasis stage, lymph node stage, tumor stage and neoplasm disease stage. According to the BRAF gene mutation status, 494 PTC patients were assigned to either the BRAF mutation group (n=237) or the BRAF wild-type group (n=257). Patients with other clinically relevant nonsilent gene mutations (such as RAS, MET, ATR and PTEN mutations) were not included in the BRAF wild-type group.

### Analysis of TIME Components

The proportion of immune-stromal components in the TIME was quantified for each sample of the BRAF mutation group (n=237) utilizing the ESTIMATE R package, manifested as three types of scores: ImmuneScore, StromalScore and ESTIMATEScore (**Supplementary Table 1**). The sum of the ImmuneScore and StromalScore is equal to the ESTIMATEScore, which represents the sum of the proportions of both in the TIME. The immune components, stromal components and combined proportion of both were positively correlated with the scores, and the higher the score was, the greater the proportion of the corresponding components. The 237 BRAF-mutant samples were divided into high and low- score groups based on the median values of the ImmuneScore, StromalScore and ESTIMATEScore.

### Association Analysis Between TIME Scores and Clinical Features

Kaplan–Meier survival analysis with the log-rank test was used to illustrate the progression-free survival (PFS) differences between the low- and high-stromal/immune/ESTIMATE score groups. The Kruskal–Wallis test or Mann–Whitney U test was applied to determine whether the above three scores were associated with neoplasm disease stage (stage), tumor stage (T classification), metastasis stage (M classification) and lymph node stage (N classification). A P value < 0.05 was considered statistically significant.

### Differential Gene Expression Analysis Between the High- and Low-Score Groups Regarding ImmuneScore, StromalScore and ESTIMATEScore

The “edgeR” R package was used to identify differentially expressed genes (DEGs) in the high-score (n = 118) and low-score (n = 119) groups. DEGs were generated from three comparisons: high vs. low StromalScore, high vs. low ImmuneScore, and high vs. low ESTIMATEScore. The cutoff criteria for identifying DEGs were *p* < 0.05 and |log2 fold change (FC)| ≥ 1. The overlapping DEGs among the above three comparisons might be significant markers for the condition of TIME.

### Functional Enrichment Analysis

Gene Ontology (GO) term and Kyoto Encyclopedia of Genes and Genomes (KEGG) pathway enrichment analyses were performed with the Metascape Database (http://metascape.org/). GO analysis mainly addressed three categories: molecular function (MF), cellular component (CC), and biological process (BP). GO terms or KEGG pathways with a p value < 0.05, a minimum overlap=3 and an enrichment factor >1.5 were considered statistically significant.

### Screening of DEGs to Predict Prognosis in BRAF-Mutated PTC

Univariate Cox regression analysis was applied to assess the overlapping DEGs associated with PFS in the BRAF-mutated PTC cohort (n = 237). The BRAF mutation-associated DEGs significantly associated with PFS were considered the candidate gene set. To validate the prognostic power of the candidate gene set, we further screened the DEGs using Kaplan–Meier survival analysis with the log-rank test to assess the PFS difference between the high-expression group and the low-expression group in the BRAF-mutated PTC cohort (n = 237). Ultimately, the above candidate genes were further filtered on the basis of Multivariate Cox regression analysis to obtain the final gene set to construct a TIME-based prognostic model in BRAF-mutated PTC.

### Construction and Estimation of a Prognostic Model in BRAF-Mutated PTC

The regression coefficient, obtained by multivariate Cox regression analysis, was combined with the expression level of the candidate gene to eventually obtain the risk score. The formulae for risk score calculation was listed as follows: Risk score = (signature1 coefficient × signature1 expression) + (signature2 coefficient × signature2 expression) + ⋯ + (signature N coefficient × signature N expression). With the median risk score as a cutoff, 237 BRAF-mutated patients were classified into high- and low-risk groups. Then, Kaplan–Meier survival analysis with the log-rank test was performed and curves were plotted to compare the PFS difference between the two groups. The predictive accuracy of the risk score and single gene was computed through the area under the curve (AUC), which was validated by time-dependent receiver operating characteristic (tROC) curve analysis for 1, 3, and 5 years. Additionally, the prognostic power of the risk score and single gene was also assessed in the BRAF wild-type cohort (n=257).

### Construction and Estimation of a Nomogram in BRAF Mutated PTC

First, the risk score and clinicopathological features (age, sex, neoplasm disease stage, metastasis stage, lymph node stage, tumor stage and radiotherapy history) were evaluated by multivariate Cox regression analysis to screen independent risk factors for PFS. Next, a nomogram was developed using the R package rms based on the former results of multivariate Cox regression analysis. Finally, ROC curve analysis was applied to evaluate the predictive power of the nomogram.

### Immune Cell Infiltration Analysis in BRAF-Mutated PTC

ImmuCellAI (http://bioinfo.life.hust.edu.cn/ImmuCellAI/#!/) is a scientific tool capable of estimating abundance and InfiltrationScore for up to 24 immune cell types from gene expression datasets (31). There were 18 T-cell subtypes (*CD4_naïve, CD8_naive, Cytotoxic, Exhausted, Tr1, nTreg, iTreg, Th1, Th2, Th17, Tfh, Central_memory, Effector_memory, NKT, MAIT, Gamma_delta_T, CD4_T and CD8_T*) and 6 other immune cell types (*B_cell, NK, Monocyte, Macrophage, Neutrophil and DC*), constituting 24 types of immune cells. Gene expression profiles of the BRAF-mutated PTC cohort were used in immune cell infiltration analysis. Two-way ANOVA was used to calculate the associations between prognostic genes or risk score and immune cell infiltration.

## Results

### Sample Information Statistics

The mRNA-seq data and corresponding clinical characteristics of patients with PTC were downloaded from the TCGA database. According to the mutation status of the BRAF gene, the PTC patients were classified into a BRAF-mutated group (n=237) and a BRAF wild-type group (n=257). The clinical data of PTC patients, including sex, age, radiotherapy history, metastasis stage, lymph node stage, tumor stage and neoplasm disease stage, were compared between the BRAF-mutated and wild-type groups. By the chi-square test, the two groups showed significant differences in tumor stage (*p*=0.014) and neoplasm disease stage (*p*=0.045) (**Table 1**). Thus, the results indicated that PTC patients with a higher tumor stage or neoplasm disease stage had a higher BRAF mutation frequency.

**Table 1:** Characteristics of the BRAF-mutated PTC patient cohort.

Characteristic	BRAF-mutated	BRAF wild-type	P value
Total	237	257	
Sex			0.656
Female	171 (72.15%)	190 (73.93%)	
Male	66 (27.85%)	67 (26.07%)	
Age			0.049
*≤*40	154 (64.98%)	188 (73.15%)	
>40	83 (35.02%)	69 (26.85%)	
Radiotherapy			0.123
Yes	133 (56.12%)	156 (60.70%)	
No	95 (40.08%)	83 (32.30%)	
Unknown	9 (3.80%)	18 (7.00%)	
Metastasis stage			0.119
M0	141 (59.49%)	135 (52.53%)	
M1+MX	96 (40.51%)	122 (47.47%)	
Lymph node stage			0.151
N0	100 (42.19%)	125 (48.64%)	
N1+NX	137 (57.81%)	132 (51.36%)	
Tumor stage			**0.014**
T1+T2	133 (56.12%)	172 (66.93%)	
T3+T4+TX	104 (43.88%)	85 (33.07%)	
Neoplasm disease stage			**0.045**
Stage1+stage2	147 (62.45%)	182 (70.82%)	
Stage3+stage4	89 (37.55%)	75 (29.18%)	

Bold values indicate statistically significant (P<0.05).

### TIME Scores Were Associated With Pathological Stage in BRAF-Mutated PTC

**Figure 2:**
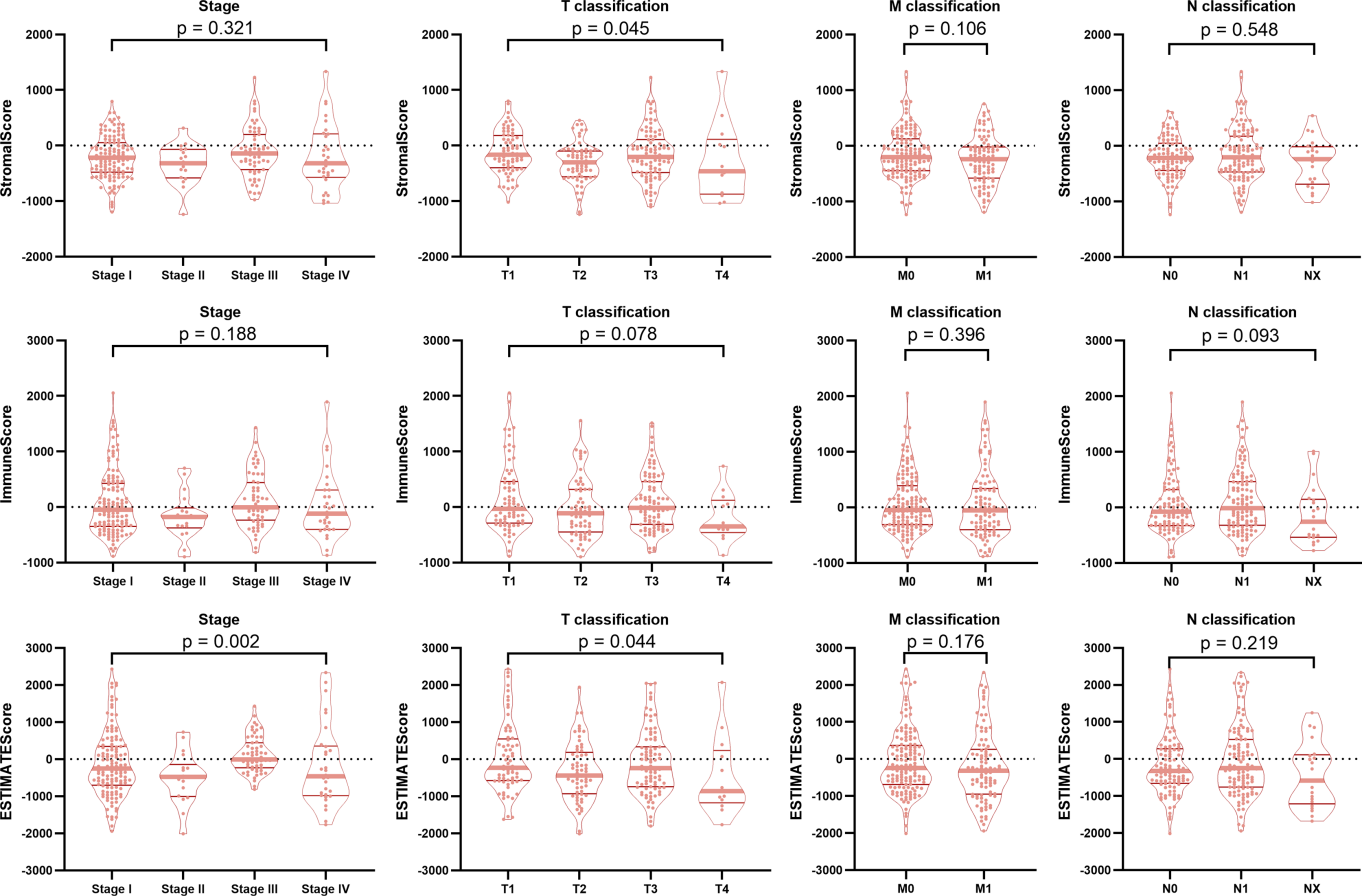
Correlation of TIME scores with clinicopathological staging characteristics. The StromalScore was correlated with the T classification of the TNM staging system (*p* = 0.045), and the ESTIMATEScore was significantly associated with the stage (*p* = 0.002) and T classification (*p* = 0.044). ImmuneScore had no correlation with the tumor stage or the T classification, M classification, or N classification of the TMN staging system (all *p* > 0.05).

To determine the relationship between TIME scores and clinical features, the neoplasm disease stage (stage), tumor stage (T classification), metastasis stage (M classification), lymph node stage (N classification) and progression-free survival (PFS) of the BRAF mutated cohort were analyzed. Although the TIME scores had no significant correlations with PFS (**Supplementary Figure 1**), the StromalScore was correlated with T classification (*p*=0.045), and the ESTIMATEScore was significantly associated with stage (*p*=0.002) and T classification (*p*=0.044) (**Figure 2**). These results implied that TIME scores were correlated with pathological stages in patients with BRAF-mutated PTC.

### Identification of Differentially Expressed Genes Associated With the TIME in BRAF-Mutated PTC

**Figure 3:**
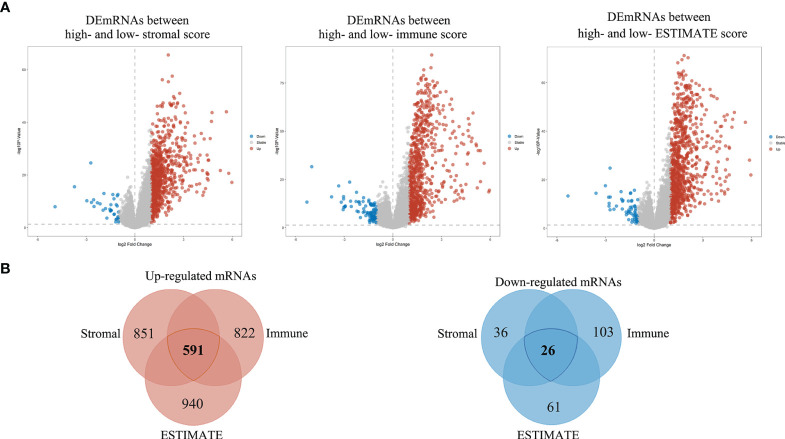
Volcano plots and Venn diagrams of DEGs in the high and low stromal/immune/ESTIMATE score groups. **(A)** Volcano plot of DEGs in the high and low stromal/immune/ESTIMATE score groups. The red, blue and gray dots represent upregulated, downregulated and unchanged genes, respectively. **(B)** Venn diagrams showing the overlapping DEGs between the high- and low-stromal/immune/ESTIMATE score groups. The total number of distinct genes in each comparison group is presented as numbers within each circle, and the number presented in the overlapping regions indicates the number of shared genes among the three comparison groups.

To ascertain the exact gene profile alterations in the TIME, three comparative analyses (high vs. low StromalScore, high vs. low ImmuneScore and high vs. low ESTIMATEScore) were carried out. The volcano plots visually showed the distribution of DEGs in the above three comparisons (**Figure 3A**). From the StromalScore (samples with high score vs. low score), a total of 887 DEGs were acquired: 851 were upregulated, and 36 were downregulated (**Supplementary Table 2**). Similarly, 925 DEGs (822 and 103 genes upregulated and downregulated, respectively) were obtained from ImmuneScore (high vs. low score). A total of 1001 DEGs were identified from the ESTIMATE score comparison (high vs. low score), comprising 940 upregulated genes and 61 downregulated DEGs. The intersection analysis displayed by the Venn diagram revealed a total of 26 downregulated genes shared by the low-score groups and 591 upregulated genes shared by the high-score groups among the StromalScore, ImmuneScore and ESTIMATEScore comparisons (**Figure 3B**). These DEGs (a total of 617 genes) were probably crucial factors affecting TIME status.

### Investigation of the Potential Functions and Pathways Associated With the TIME in BRAF-Mutated PTC

**Figure 4:**
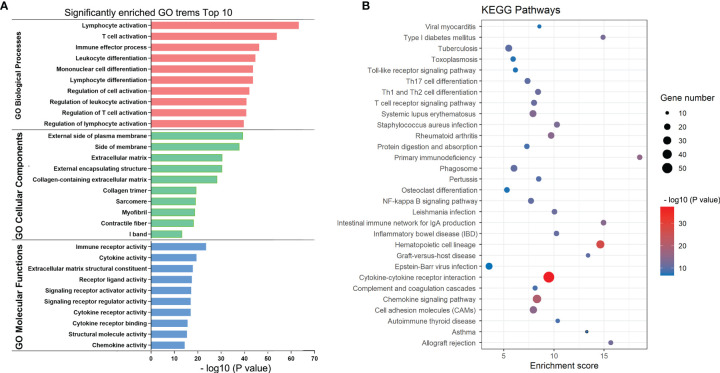
KEGG and GO enrichment plots of DEGs. **(A)** GO terms enriched with the DEGs in the biological process (BP), cellular component (CC) and molecular function (MF) categories. The Y-axis shows the different subcategories and the X-axis shows the log transformed *p* values. **(B)** KEGG pathways enriched with the DEGs. The y-axis shows the different pathway entries and pathway names, the x-axis shows the enrichment scores, and the bubbles indicate the numbers of genes.

To elucidate TIME-related potential biological functions and pathways, Gene Ontology (GO) term and KEGG pathway enrichment analyses were performed on the above 617 DEGs. Based on the Metascape database, we examined a total of 2084 GO assignments, including 1750 BPs, 194 MFs, and 140 CCs. In the BP category, most genes were involved in T-cell activation, regulation of lymphocyte activation, immune effector processes, leukocyte differentiation and mononuclear cell differentiation. In the MF category, most genes may play roles in immune receptor activity, cytokine activity, extracellular matrix structural components, receptor ligand activity and signaling receptor activator activity. In the CC category, most genes were associated with the external side of the plasma membrane, side of the membrane, extracellular matrix, external encapsulating structure and collagen-containing extracellular matrix. The top 10 significantly enriched GO terms are shown in **Figure 4A**. Furthermore, KEGG pathway analysis revealed that 84 pathways, such as cytokine–cytokine receptor interaction, chemokine signaling pathway, primary immunodeficiency and cell adhesion molecules (CAMs), were significantly enriched (**Figure 4B**). According to the p values in the GO and KEGG enrichment analyses, immune-related activities were the most significantly enriched terms and pathways, consistent with the TIME phenotype. Thus, these results provided key insights into the mechanism of BRAF-mutated PTC from the perspective of the TIME.

### Establishment of a Prognostic Model for PTC Patients With BRAF Mutation

**Figure 5:**
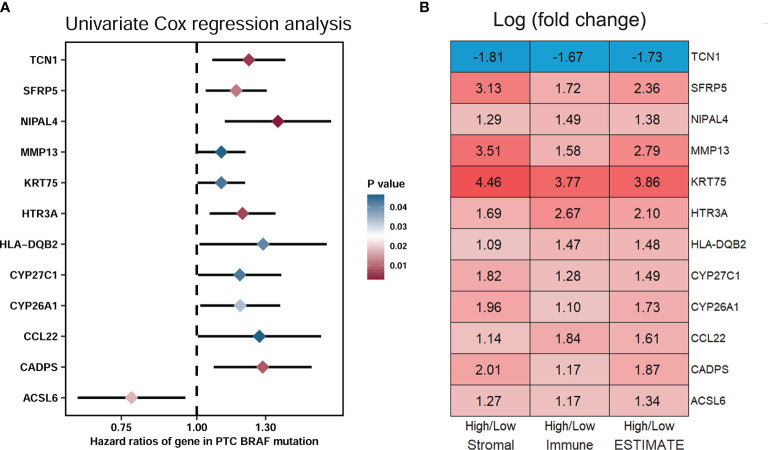
Screening of DEGs related to significant prognosis in BRAF-mutated PTC. **(A)** The forest plot showed that 12 DEGs (*TCN1, SFRP5, NIPAL4, MMP13, KRT75, HTR3A, HLA-DQB2, CYP27C1, CYP26A1, CCL22, CADPS* and *ACSL6*) were significantly associated with the PFS of PTC patients with BRAF mutation (p < 0.05) by univariate Cox regression analysis. **(B)** Module diagram showing the log2 fold changes (FCs) in the twelve selected DEGs between the high vs. low stromal/immune/ESTIMATE score groups.

**Figure 6:**
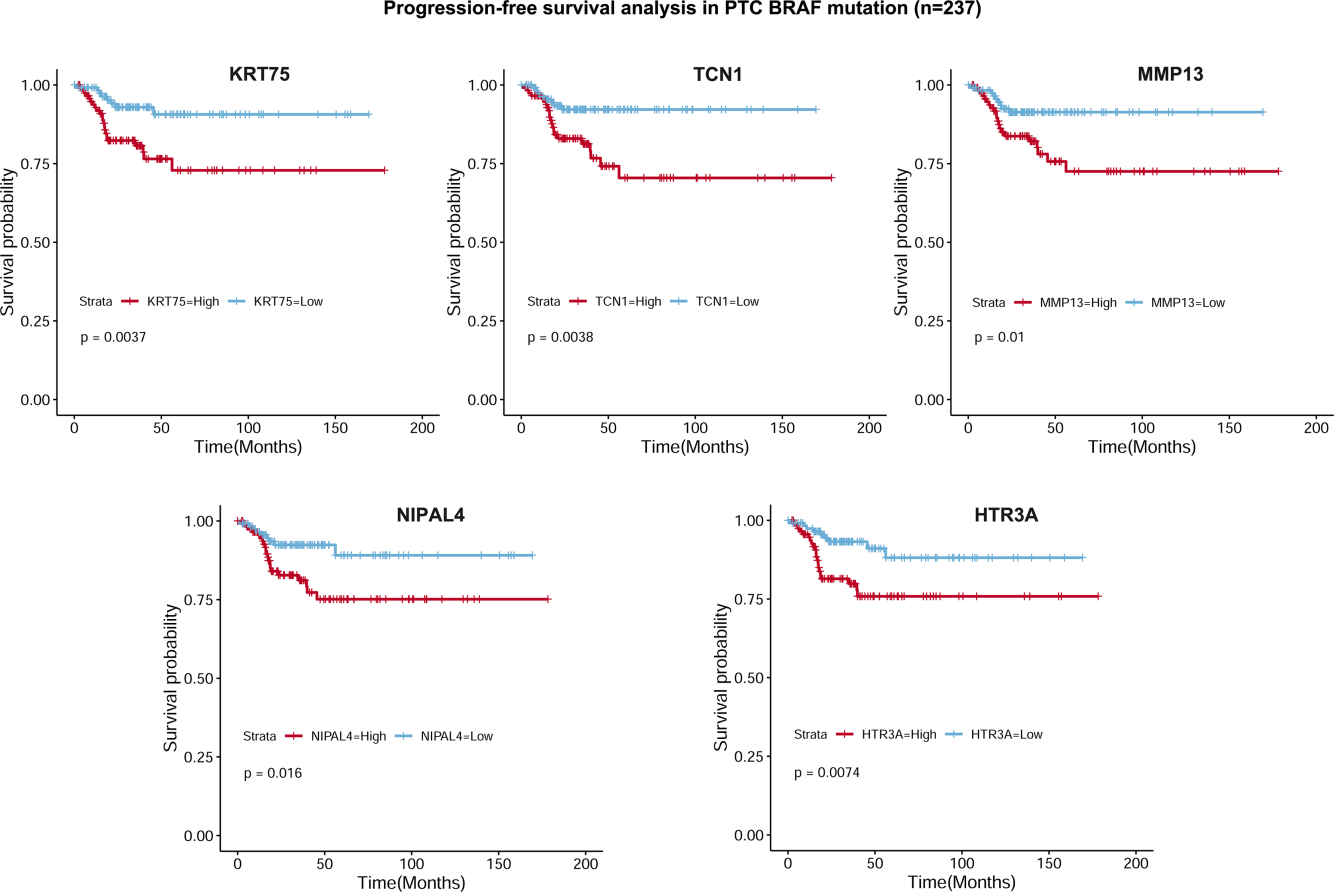
Kaplan–Meier survival curves based on 5 DEGs. Progression-free survival analysis revealed that higher expression levels of *KRT75, TCN1, MMP13, NIPAL4* and *HTR3A* were related to shorter PFS in PTC patients with BRAF mutation (*p* < 0.05).

To select the qualified DEGs for developing a prognostic prediction model, the above 617 DEGs were analyzed as variables with univariate Cox regression analysis. Twelve DEGs (*TCN1, SFRP5, NIPAL4, MMP13, KRT75, HTR3A, HLA-DQB2, CYP27C1, CYP26A1, CCL22, CADPS* and *ACSL6*) were significantly associated with PFS in BRAF-mutated PTC patients (**Figure 5A**). The log2 fold change (FC) of these DEGs in the comparisons between high- and low-stromal/immune/ESTIMATE score was shown in **Figure 5B**. Then, the 12 genes were further screened using Kaplan–Meier survival analysis (**Supplementary Figure 2**), and the results revealed that higher expression levels of *KRT75, TCN1, MMP13, NIPAL4* and *HTR3A* were related to the shorter PFS times of patients with BRAF mutation (*p*<0.05) (**Figure 6**). Ultimately, these five genes were further filtered on the basis of multivariate Cox regression analysis, and only *HTR3A* (HR=1.151 (95% CI: 1.001-1.325), *p*=0.048) and *NIPAL4* (HR=1.268 (95% CI: 1.023-1.571), *p*=0.030) were statistically significant.

**Figure 7:**
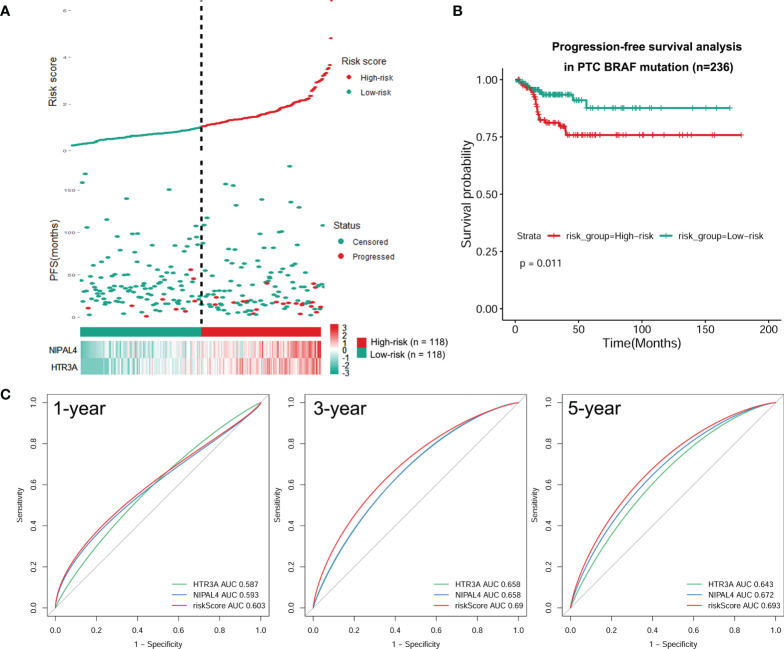
Construction of a risk model for BRAF-mutated PTC. **(A)** Risk score based on survival status, 2 DEG signatures and expression heatmap for each patient with BRAF-mutated PTC. Elevated expression levels of HTR3A/NIPAL4 and the disease progression rate in patients with BRAF-mutated PTC were positively correlated with high risk scores. **(B)** PFS difference between high- and low-risk groups using the Kaplan–Meier curve. The Kaplan–Meier survival curves revealed that the high-risk group had a lower PFS rate than the low-risk group (*p* < 0.05). **(C)** The tROC curve of the prognostic risk model compared to the single-variable models. The tROC curve at 1, 3 and 5 years revealed that the AUC for the risk score was higher than that for any single gene.

**Figure 8:**
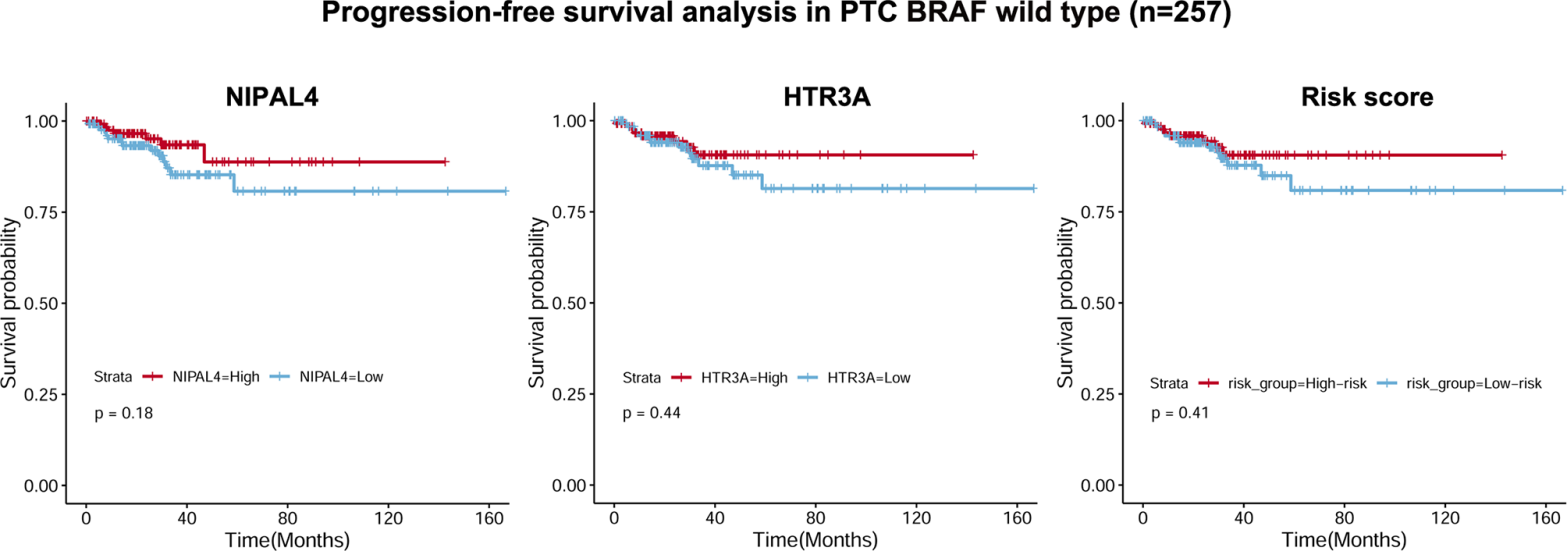
Correlations of NIPAL4 expression, HTR3A expression and risk score with PFS in BRAF wild-type PTC (n = 257). The Kaplan–Meier survival curves showed that PFS did not differ significantly (all *p >* 0.05) between the low/high NIPAL4 groups, the high/low HTR3A groups and the high/low risk score groups in the BRAF wild-type PTC cohort.

We developed a prognostic model combining the abovementioned 2 DEGs (*HTR3A and NIPAL4*) through multivariate Cox regression analysis. The risk score for every patient was calculated as follows: Risk Score = [Expression level of HTR3A * (0.2289)] + [Expression level of NIPAL4 * (0.2970)]. Then, using the median risk score as the threshold, the BRAF-mutated cohort was divided into the high-risk (n=118) and low-risk groups (n=118). The progressive disease rate of BRAF-mutated patients increased with the prognostic risk score, and the heatmap showed that the expression of *HTR3A* and *NIPAL4* was upregulated in the high-risk group (**Figure 7A**). The high-risk group had less favorable PFS than the low-risk group, as shown by the Kaplan–Meier curve (*p*<0.05) (**Figure 7B**). Furthermore, the ROC curve revealed that the risk score had a larger area under the curve (AUC) than a single gene (**Figure 7C**), which illustrated that the risk score could yield better accuracy to predict the PFS of BRAF-mutated PTC patients. Moreover, PFS analyses of high/low risk scores, high/low *HTR3A* and high/low *NIPAL4* were also performed in BRAF wild-type (n=257) PTC patients, which demonstrated no statistical significance (**Figure 8**). Taken together, the risk score model composed of *HTR3A* and *NIPAL4* presented a good predictive ability in PTC patients with BRAF mutation.

### Construction of a Prognostic Nomogram for PTC Patients With BRAF Mutation

**Figure 9:**
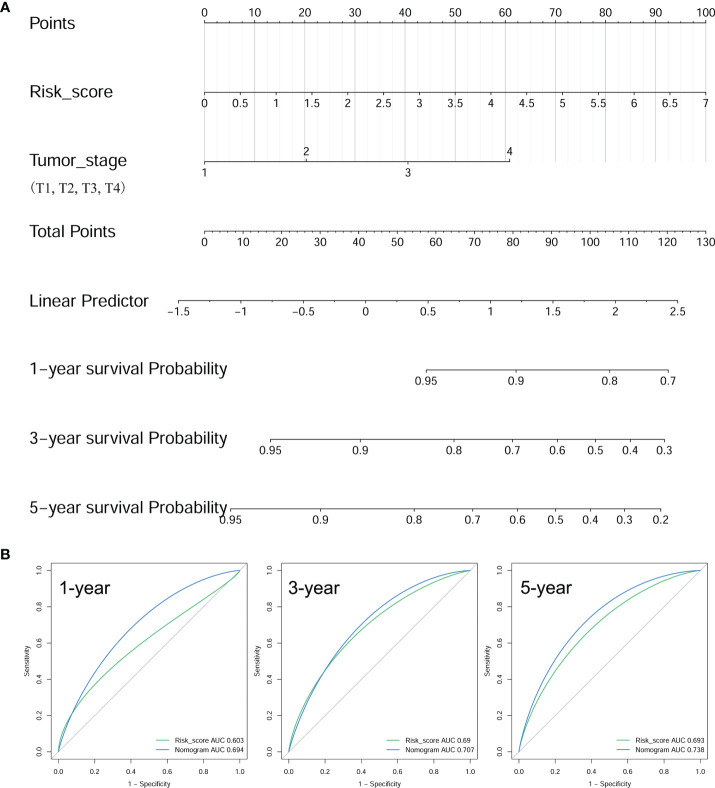
Construction of a prognostic nomogram. **(A)** Nomogram to predict the 1-, 3-, and 5-year PFS of BRAF-mutated PTC based on risk score and tumor stage. **(B)** The tROCs for 1-, 3-, and 5-year PFS predictions for the nomogram and risk score. The tROC curve showed that the AUCs of the nomogram were higher at 1, 3 and 5 years (0.694, 0.707 and 0.738, respectively) than those of the risk score model.

To elevate the predictive ability of the risk score model, a nomogram was generated by combining the risk score and clinical parameters of patients with BRAF-mutated PTC. The risk score and relevant clinical data (age at diagnosis, sex, neoplasm disease stage, metastasis stage, lymph node stage, tumor stage and radiotherapy history) were analyzed by multivariate Cox regression analysis (**Table 2**). The results verified that risk score (HR=1.614 (95% CI: 1.199-2.173), *p*=0.002) and tumor stage (HR=1.838 (95% CI: 1.228-2.750), *p*=0.003) were independent risk factors for PFS in the BRAF-mutated cohort. These two factors verified by the multivariate analysis were included in the prognostic nomogram (**Figure 9A**). To elucidate the putative advantages of the nomogram, we performed tROC curve analysis. Compared with the risk score model, the AUCs of the nomogram were higher at 1, 3 and 5 years (0.694, 0.707 and 0.738, respectively; **Figure 9B**). These results suggested the improved clinical utility of the nomogram in the BRAF-mutated PTC cohort.

**Table 2:** Multivariate analysis of progression-free survival in the BRAF-mutant PTC cohort.

Variable	HR	95% CI	P value
Risk score	1.614	1.199-2.173	**0.002**
Age at diagnosis	0.977	0.940-1.017	0.257
Sex	0.995	0.437-2.262	0.990
Neoplasm disease stage	1.509	0.905-2.515	0.115
Metastasis stage	1.835	0.884-3.812	0.103
Lymph node stage	1.139	0.598-2.168	0.692
Tumor stage	1.838	1.228-2.750	**0.003**
Radiotherapy	1.241	0.522-2.952	0.624

Bold values indicate statistically significant (P<0.05).

### Features of Infiltrating Immune Cells in BRAF-Mutated PTC

**Figure 10:**
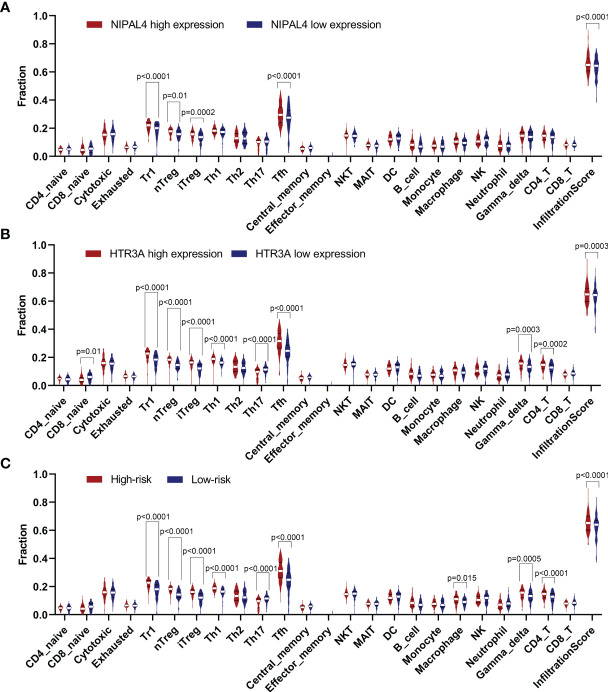
Violin plot showing the compression of each immune cell type in the low/high NIPAL4 groups **(A)**, low/high HTR3A groups **(B)** and low/high risk groups **(C)**. The high-risk/HTR3A/NIPAL4 groups had a higher degree of immune cell infiltration (*p* < 0.05), suggesting that the risk score, HTR3A and NIPAL4 are involved in the prognosis of BRAF-mutated PTC by interfering with immune cells.

As risk score, HTR3A and NIPAL4 were associated with the poor prognosis of PTC patients with BRAF mutation, and we further evaluated the correlation between immune cells and the above three factors. We observed that the Tr1, nTreg, iTreg and Tfh cell numbers were increased in the high-NIPAL4 group and that the Tr1, nTreg, iTreg, Th1, Tfh, gamma delta T, and CD4^+^ T cell numbers were increased in the high-HTR3A group and high-risk group; conversely, the Th17 cell number was decreased. In addition, macrophages were increased only in the high-risk group, while CD8^+^ naive cells decreased only in the high-HTR3A group. The infiltration scores between the low-NIPAL4 and high-NIPAL4 groups, the low-HTR3A group and the high-HTR3A group, and the low- and high-risk groups were all statistically significant (*p*<0.05) (**Figure 10**). These findings suggested that HTR3A expression and NIPAL4 expression were likely to be involved in BRAF-mutated PTC by interfering with immune cells.

## Discussion

The existence of BRAF mutations frequently occurs in PTC, and its presence is thought to be associated with the aggressive progression of PTC (6, 32), but its role as an independent prognostic predictor is controversial. Existing risk stratification systems, such as ATA risk stratification and TNM staging, fail to adequately predict clinical outcomes in individual PTC patients. Many studies have demonstrated that TIME-related genes can interfere with or predict tumor prognosis, but to date, no single prognostic gene or multigene prognostic model for BRAF-mutated PTC has been reported. In this study, we particularly focused on establishing a TIME-related prognostic model in BRAF-mutated PTC populations, which was innovative and disparate from previous studies. Ultimately, we successfully constructed a nomogram by combining the risk score of prognostic TIME-related genes and clinical parameters to predict PFS in PTC patients with BRAF mutation.

Based on comprehensive analysis, we identified two optimal TIME-related genes, *HTR3A* and *NIPAL4*, as potential prognostic biomarkers of BRAF-mutated PTC. The 5-HT3 receptor is the only ligand-gated cation channel among the 5-HT (5‐hydroxytryptamine) receptor family, consisting of five subunits, HTR3A-E. HTR3A is the only subunit capable of forming functional homopentameric channels. HTR3A has been demonstrated to participate in various biological processes, such as organismal energy homeostasis, interneuron migration and arrhythmias (33–35). Several recent papers have discussed the effects of HTR3A on tumor cells, which plays a role in tumor progression in specific types of cancer (36, 37). For example, Tone et al. found that higher HTR3A expression was significantly associated with pathological stage, lymph node metastasis, lymphovascular invasion and recurrence in lung adenocarcinoma and further suggested that HTR3A promotes the aggressiveness of lung adenocarcinoma through ERK1/2 phosphorylation (36). Tang et al. found that HTR3A may contribute to the development of colorectal carcinoma (CRC) and confirmed that the expression of some Bcl-2 family proteins, including BAX, BCL-2 and BAD proteins, was mainly regulated by HTR3A and participated in CRC cell cycle progression, cell proliferation and apoptosis (37). The present study confirmed that upregulation of HTR3A was associated with shorter PFS in BRAF-mutated PTC, supporting a tumor-promoting role of HTR3A and a potential role in predicting prognosis. NIPA4, also called ichthyin, is a membrane protein that is thought to be a magnesium transporter with a structure homologous to G protein-coupled receptors that plays a pivotal role in epidermal lipid metabolism, but the mechanism remains unclear (38, 39). To date, only a limited number of mutations in the NIPAL4 gene have been described to be correlated with autosomal recessive congenital ichthyosis (ARCI) (40–42). At present, no reports concerning these two genes have been published in PTC; thus, their role in BRAF-mutated PTC needs further investigation.

Tumor-infiltrating immune cells (TICs) play a pivotal role in the TIME, and their number and distribution show clinicopathological significance in predicting prognostic and therapeutic efficacy (43–46). A recent study explored seven TIME-related genes in HCC, and the results demonstrated that high expression levels of *BIRC5, IL11, IL17D, SPP1* and *FGF13* were associated with a low overall survival (OS) rate and found a strong positive correlation between infiltration of five immune cell types (macrophages, neutrophils, CD8^+^ T cells, B cells and dendritic cells) and the expression of these seven genes (47). Shichao Zhang et al. found that the abundance of TICs (such as monocytes, resting DCs and M2 macrophages) was associated with poor OS in gastric cancer patients and that the expression levels of most hub TIME-related genes, especially *C3AR1, F2R, PLXNC1, CYSLTR1, GHR, GLP2R* and *RNASE2*, were significantly positively correlated with the abundance of TICs (48). Recently, Rujia Qin et al. identified lactotransferrin (LTF) as a key TIME-related gene associated with the disease course of PTC and found that nine types of immune cells (CD8^+^ T cells, B cells, aDCs, macrophages, mast cells, NK cells, Tfh cells, Treg cells and DCs) were significantly correlated with LTF expression (24). These studies suggest that TIME-related genes can affect tumor prognosis by regulating immune cell infiltration. The results of our study also support this view. Our work demonstrated that HTR3A and NIPAL4 expression were associated with prognosis and tumor-infiltrating immune cells. Further immune infiltration analyses showed that high HTR3A expression presented high immune infiltration of Tr1, nTreg, iTreg, Th1, Tfh, gamma delta T cells, and CD4+ T cells, and samples with high NIPAL4 expression presented high immune infiltration of Tr1, nTreg, iTreg and Tfh cells in BRAF-mutated PTC. In addition, the immune infiltration level in tumors in the high-risk score group tended to be increased. Our study contributes novel insights into the interactions between tumor cells and infiltrating immune cells, confirming that HTR3A and NIPAL4 may be involved in BRAF-mutated PTC by interfering with immune cells.

Nomograms have shown promising results in predicting clinical risk characteristics and prognosis in certain cancers and have been extensively applied (49, 50). For example, Guolin Wu et al. used the risk score method to identify 3 genes associated with adverse prognosis of pancreatic adenocarcinoma (PAAD), *ERAP2*, *CKLF* and *EREG*, and constructed a nomogram based on clinical features and risk score for individualized prognosis prediction (51). The study conclusively demonstrated that the nomogram is a noninvasive predictor and can be used as a progression-related indicator of OS in PAAD patients (51). Congkuan Song et al. found that four TIME-related genes (*OAS1, WFDC2, MS4A1 and MAL*) were associated with the prognosis of lung adenocarcinoma (LUAD) by studying 535 lung tumor tissues and 59 normal lung tissues (52). They then constructed a nomogram predicting patient outcomes in the TCGA database based on the four–TIME-associated–gene signature (risk score) and a clinical feature (TNM stage) (52). The results confirmed that compared with the ideal model, the calibration curves of the development set, the internal validation set and the external validation set of the model established in this study had good consistency, proving that the nomogram was reliable and stable for predicting the outcome of LUAD patients (52). In our work, a nomogram was constructed by integrating the risk score of two TIME-related genes and selected clinical parameters in PTC patients with BRAF mutation. After validation by ROC curve analysis, we found that the clinical utility of the nomogram in the BRAF-mutated PTC cohort was improved compared with that of the risk score model. It should be noted that our model includes a lower number of genes, indicating lower costs and, consequently, increased clinical utility.

This study also has several limitations. First, the sample size was limited. Second, each case was retrospectively selected, which could potentially introduce bias. Finally, we collected 4 datasets involving BRAF-mutated PTCs in the GEO database (GSE48953 (20 samples), GSE27155 (51 samples), GSE53157 (7 samples), GSE54958 (25 samples)), but none of them had clinical information such as PFS, so currently we have no more valid external independent datasets to further verify our model. These limitations need to be improved upon in subsequent studies. Despite the limitations noted above, this study still has its strengths and innovations. In summary, the present study is the first to screen for prognostic TIME-related genes associated with BRAF mutation status and combine them to construct a risk score model and a nomogram. Although concrete indications and strategies for using our model for individualized prediction of PTC patients with BRAF mutation need to be defined, the potential value of this model in predicting PFS in BRAF-mutated PTC should be acknowledged. It is expected that the prognostic use of this model will add a spatial dimension to the developing current risk stratification system, which may have significant ramifications for the current clinical treatment of thyroid neoplasms.

## Data Availability Statement

The original contributions presented in the study are included in the article/**Supplementary Material**. Further inquiries can be directed to the corresponding authors.

## Author Contributions

WT and YS conceived the study idea. XJ, QL, YiH, and BZ collected the data to be analyzed. ZM, DX, and YX performed the data analysis and produced the results. YX, YuH, and WT wrote and revised the manuscript. All authors contributed to the article and approved the submitted version.

## Funding

This work was supported by Scientific Research Fund of Chengdu Medical College (CYZ18-12 and CYZ18-24), Project of Science & Technology Department of Sichuan Province (2020JDRC0134), Medical Youth Innovation Research Project of Sichuan Province (Q21076), Scientific Research Project of Medicine Department of Sichuan Province (S18001), Health Commission of Sichuan Province (20PJ227), Scientific Research Project of Medicine Department of Chengdu City (2021051), and Scientific Research Project of China Baoyuan Investment Co., Ltd (CBYI202105).

## Conflict of Interest

The authors declare that the research was conducted in the absence of any commercial or financial relationships that could be construed as a potential conflict of interest.

## Publisher’s Note

All claims expressed in this article are solely those of the authors and do not necessarily represent those of their affiliated organizations, or those of the publisher, the editors and the reviewers. Any product that may be evaluated in this article, or claim that may be made by its manufacturer, is not guaranteed or endorsed by the publisher.

## Supplementary Material

Supplementary Table 1 ImmuneScore, StromalScore and ESTIMATEScore of the BRAF mutation group.

Supplementary Table 2 DEGs in the high and low stromal/immune/ESTIMATE score groups of the BRAF mutation group.

Supplementary Figure 1 Correlations of TME scores with PFS in BRAF-mutated PTC. Kaplan–Meier survival analysis for BRAF-mutated samples grouped by high or low StromalScore (*p*=0.9), ImmuneScore (*p*=0.34) and ESTIMATEScore (*p*=0.63), as determined by comparison with the median value.

Supplementary Figure 2 Kaplan–Meier survival curves based on 12 DEGs. Progression-free survival analysis revealed that higher expression levels of 5 DEGs (*KRT75, TCN1, MMP13, NIPAL4 and HTR3A*) were related to shorter PFS in BRAF-mutated PTC (all *p* < 0.05), while the expression levels of the remaining 7 DEGs (*SFRP5, HLA-DQB2, CYP27C1, CYP26A1, CCL22, CADPS* and *ACSL6*) had no significant correlation with PFS in BRAF-mutated PTC (all *p*>0.05).
