# Supplementary figures and images for: Construction of a Tumor Immune Microenvironment-Related Prognostic Model in BRAF-Mutated Papillary Thyroid Cancer
Source: Front Endocrinol (Lausanne). 2022 Jun 8;13:895428. doi: 10.3389/fendo.2022.895428 (PMC9215106; doi:10.3389/fendo.2022.895428)

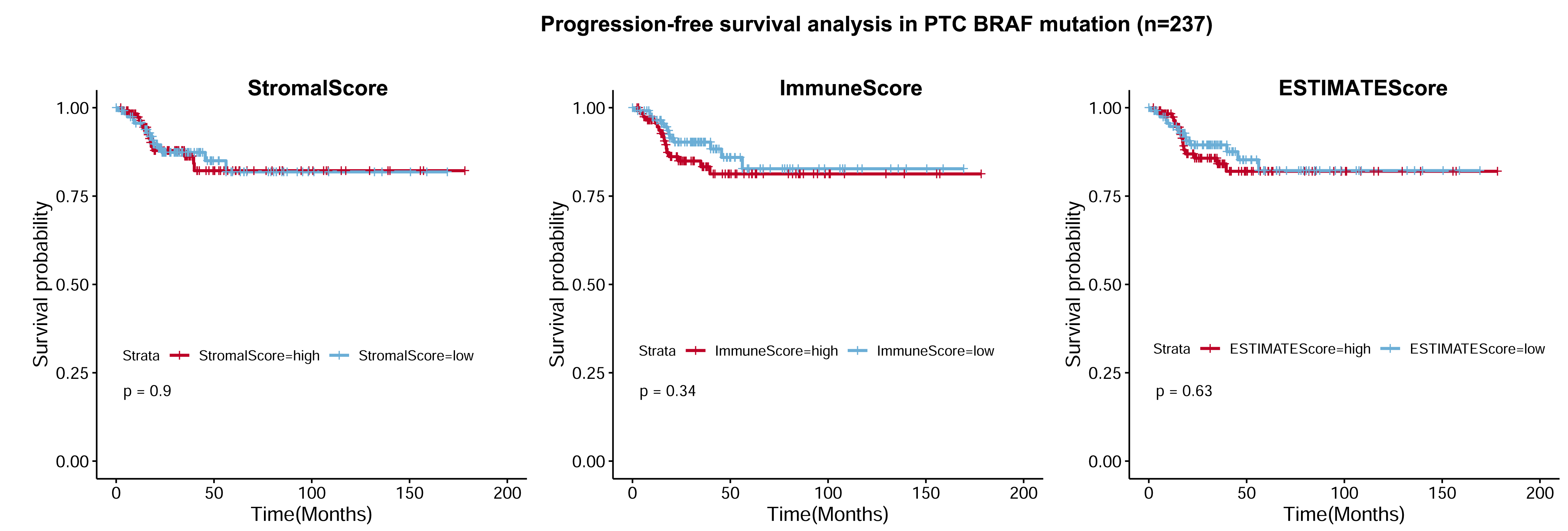

Supplement: Supplementary Figure 1 — Correlations of TME scores with PFS in BRAF-mutated PTC. Kaplan–Meier survival analysis for BRAF-mutated samples grouped by high or low StromalScore (p=0.9), ImmuneScore (p=0.34) and ESTIMATEScore (p=0.63), as determined by comparison with the median value. [file Image_1.tif]

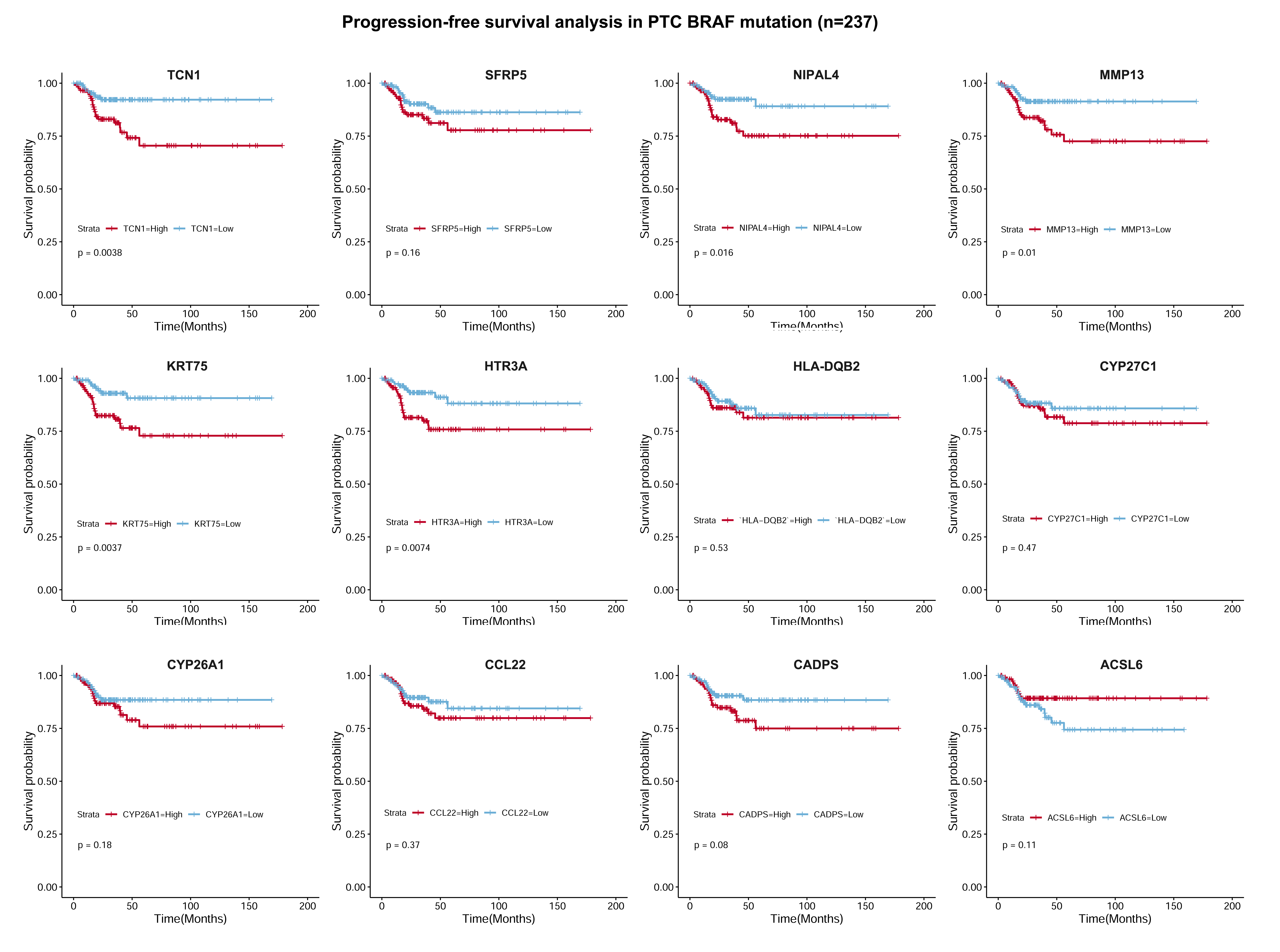

Supplement: Supplementary Figure 2 — Kaplan–Meier survival curves based on 12 DEGs. Progression-free survival analysis revealed that higher expression levels of 5 DEGs (KRT75, TCN1, MMP13, NIPAL4 and HTR3A) were related to shorter PFS in BRAF-mutated PTC (all p < 0.05), while the expression levels of the remaining 7 DEGs (SFRP5, HLA-DQB2, CYP27C1, CYP26A1, CCL22, CADPS and ACSL6) had no significant correlation with PFS in BRAF-mutated PTC (all p>0.05). [file Image_2.tif]
